# Profiling of genetic determinants required for fitness of community-associated methicillin-resistant *Staphylococcus aureus* in human blood

**DOI:** 10.1128/spectrum.03585-25

**Published:** 2026-04-21

**Authors:** Nader Abdelmalek, Sally W. Yousief, Martin S. Bojer, John E. Olsen, Salvatore Rubino, Bianca Paglietti

**Affiliations:** 1 Department of Biomedical Sciences, University of Sassari 390773, Sassari, Italy; 2 Unit of Microbiology and Virology, University Hospital of Sassari, Sassari, Italy; 3 Department of Veterinary and Animal Sciences, Faculty of Health and Medical Sciences, University of Copenhagen 53139, Copenhagen, Frederiksberg, Denmark; University of Calgary, Calgary, Alberta, Canada

**Keywords:** *Staphylococcus aureus*, TraDIS, bloodstream infection, fitness, metabolism

## Abstract

Community-associated methicillin-resistant *Staphylococcus aureus* (CA-MRSA) is a leading cause of bacteremia, yet the genetic basis for its success in this hostile environment remains poorly defined. In this study, we employed transposon-directed insertion site sequencing (TraDIS) to map the fitness landscape of CA-MRSA strain USA300 JE2 through a genome-wide screening in fresh, immunocompetent human blood. We identified 76 genes required for fitness, including genes involved in respiratory and central carbon metabolisms, heme detoxification, and *de novo* purine biosynthesis. As validation of fitness genes, competition assays confirmed that individual disruption of *purA*, *purB*, *fbp*, *hssR*, or *aroA2* significantly reduced bacterial fitness in blood. Conversely, inactivation of specific regulators, such as the *saeRS* two-component system, the alternative sigma factor σ^B^, and adhesins, including *fnbA* and *clfA*, conferred a competitive advantage. These findings provide a genome-scale map of *S. aureus* fitness requirements in a physiologically relevant blood model, offering a platform for further investigation of bacterial adaptation to the intravascular environment.

**IMPORTANCE:**

Understanding how *Staphylococcus aureus* maintains fitness in the human bloodstream is essential for explaining its success as an invasive pathogen. This study provides a comprehensive, genome-wide definition of the genes that enable *S. aureus* to remain competitive in blood, revealing the key physiological requirements for adaptation to this challenging environment. By identifying genetic functions whose disruption impairs fitness, our findings highlight the specific pathways that sustain adaptation and competitiveness under host-imposed stress. Extending previous genome-scale investigations conducted in other infection niches, this study emphasizes the importance of physiological context in shaping bacterial fitness and identifies conserved cross-fitness determinants shared among *S. aureus* lineages. These insights advance our current understanding of how *S. aureus* adapts to the bloodstream and strengthen the foundation for future functional and comparative studies on staphylococcal pathophysiology.

## INTRODUCTION

*Staphylococcus aureus* is an opportunistic pathogen capable of causing a wide range of infections, from superficial skin abscesses to life-threatening systemic conditions, such as bacteremia and endocarditis (1). Among these, bloodstream infections pose a particularly severe clinical challenge to the healthcare system. *S. aureus* is the leading cause of invasive infection-related mortality worldwide, with case fatality rates ranging from 15% to 30% and an estimated 300,000 deaths annually (2). A substantial proportion of these fatalities is attributed to methicillin-resistant *S. aureus* (MRSA), which exhibits elevated resistance to conventional antibiotics and offers limited therapeutic options, resulting in poorer patient outcomes (3).

A major contributor to the pathogenicity of *S. aureus* is its ability to survive and proliferate within the human bloodstream, an environment characterized by robust immune defenses and limited nutrient availability (4). Upon entering the bloodstream, *S. aureus* encounters multiple host defense mechanisms, including neutrophil-mediated immune surveillance, complement activation, and nutritional immunity, which restrict microbial fitness (5). In response to these barriers, *S. aureus* has evolved a diverse array of strategies to evade immune clearance and establish invasive infections (4, 5). However, the full repertoire of genetic determinants required for fitness in the bloodstream remains incompletely defined.

Recent advances in functional genomics have facilitated comprehensive investigations into the fitness of *S. aureus* and other bacteria across diverse host environments (6–9). Approaches such as transcriptomics, genome-wide association studies, and high-throughput transposon mutant library screens have shed light on *S. aureus* adaptation within the bloodstream (10–13). Among these, transposon-directed insertion site sequencing (TraDIS) has emerged as a particularly powerful method for genome-wide fitness profiling (14). While TraDIS has been applied to livestock-associated MRSA (LA-MRSA) in fresh human blood (12), and Tn-seq recently to community-associated MRSA (CA-MRSA) in commercially sourced, immune-depleted blood (15), a genome-wide investigation has not yet been applied to CA-MRSA in fresh, immune-competent human blood.

In this study, we employed TraDIS to comprehensively investigate both fitness-enhancing and fitness-reducing genetic determinants involved in CA-MRSA fitness and metabolic adaptation in human blood. These findings provide novel insights into the molecular mechanisms underlying MRSA persistence in the bloodstream and establish a foundation for the development of targeted therapeutic strategies.

## RESULTS

### Assessment of bottleneck effects during TraDIS screening in human blood

**Fig 1:**
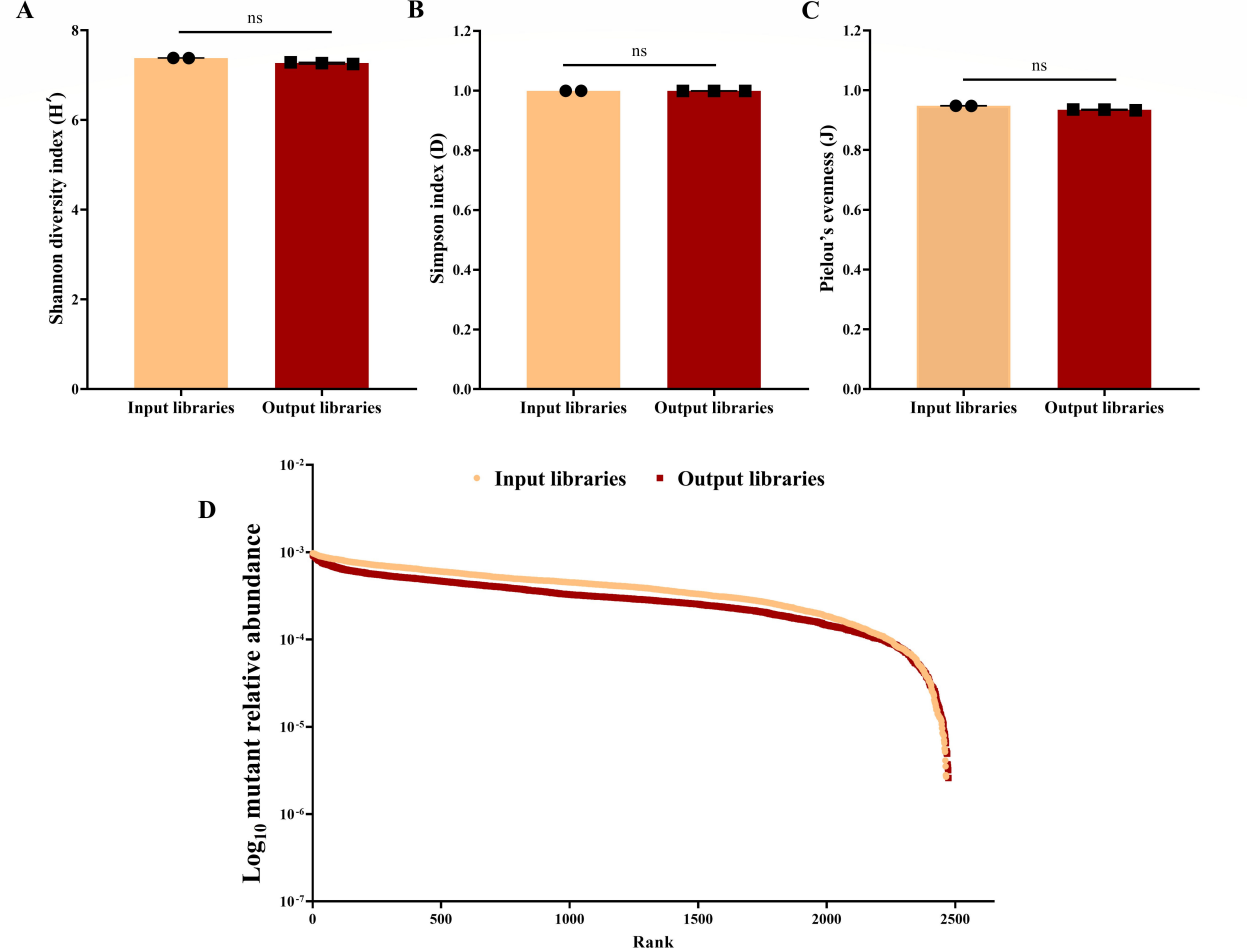
Diversity and rank-abundance of transposon mutant libraries. (**A**) Shannon index, (**B**) Simpson index, and (**C**) Pielou’s evenness for input (orange) and output (red) libraries. Points represent biological replicates, and bars indicate means. *P* values were determined by the Mann–Whitney U test; asterisks indicate adjusted significance levels (ns = not significant). (**D**) Rank-abundance curve showing the relative abundance of each mutant ranked from most to least abundant in input (orange) and output (red) libraries; curves largely overlap, indicating conservation of mutant representation.

To investigate the genetic determinants of *S. aureus* fitness in human blood, a high-density USA300 JE2 transposon mutant library, containing approximately 2 × 10⁹ colony-forming units (CFU)/mL (16), was seeded into fresh blood from three independent human volunteers. Following the blood challenge, the recovered populations (approximately 10⁹ CFU/mL from each replicate) were processed for genomic DNA extraction and sequencing (Table S1). Principal component analysis (PCA) of insertion counts revealed clear separation among blood replicates, input (16), and outgrowth control samples (17), with high consistency across blood replicates (Fig. S1). TraDIS analysis of libraries recovered after growth showed preserved insertional richness, with approximately 400,000 unique insertion sites comparable to the input library (Table S1). To quantitatively assess population bottlenecks, we calculated Shannon, Simpson, and Pielou’s evenness indices from normalized gene-level read counts. All three indices confirmed that neither diversity nor evenness was significantly reduced in the output libraries, indicating that the overall complexity was largely maintained (Fig. 1A through C). The rank-abundance curve further showed nearly overlapping input and output distributions (Fig. 1D), confirming that the relative abundance of most mutants was conserved. The effective population size (*N*ₑ) estimated from the inverse Simpson index remained at 70%–84% of the input, averaging 77%, consistent with a moderate bottleneck.

### Identification of genes of *S. aureus* with decreased fitness in human blood environment

To identify genes required for bacterial fitness in human blood, we first compared the transposon insertion profiles after 24 h of exposure to human blood with the input transposon library, revealing 146 genes significantly depleted (log₂ fold change [Log_2_FC] ≤ –2, q-value < 0.01, log count per million [logCPM] > 2, mean control reads > 150) (Table S2A). To distinguish genes specifically required for fitness in blood from those broadly essential for growth under laboratory conditions, we next compared the blood data set with two previously generated outgrowth controls in brain heart infusion (BHI) broth (Table S2B) (17). TraDIS analysis identified 76 genes significantly depleted in blood (Log_2_FC ≤ –2, q-value < 0.01, logCPM > 2, mean control reads > 150) but not during growth in BHI, indicating that disruption of these genes specifically impaired fitness in blood. These genes included many involved in menaquinone-dependent respiration, nucleotide metabolism, and metal regulation (Table 1; Fig. S2).

**TABLE 1:** Predicted genes required for *S. aureus* USA300 JE2 fitness during survival in human blood^*a*^

Locus tag	Gene name	Description	Log_2_FC	q-value
Menaquinone biosynthesis				
SAUSA300_1737	*menE*	O-succinylbenzoate-CoA ligase	−5.31	8.04 × 10^−29^
SAUSA300_0948	*menB*	Naphthoate synthase	−4.22	4.04 × 10^−18^
SAUSA300_1360	*ubiE*	Menaquinone biosynthesis methyltransferase ubiE	−4.05	3.89 × 10^−24^
SAUSA300_0946	*menD*	2-Succinyl-6-hydroxy-2,4-cyclohexadiene-1-carboxylic acid synthase/2-oxoglutarate decarboxylase	−3.65	4.18 × 10^−19^
SAUSA300_0945	*menF*	Isochorismate synthase family protein	−3.61	4.90 × 10^−17^
SAUSA300_1735	*menC*	O-succinylbenzoic acid synthetase	−3.31	4.85 × 10^−08^
Chorismate biosynthesis				
SAUSA300_1357	*aroC*	Chorismate synthase	−4.60	3.40 × 10^−17^
SAUSA300_1355	*aroA*	3-Phosphoshikimate 1-carboxyvinyltransferase	−4.56	1.28 × 10^−24^
SAUSA300_0787	*aroD*	3-Dehydroquinate dehydratase, type I	−4.25	1.49 × 10^−13^
SAUSA300_1356	*aroB*	3-Dehydroquinate synthase	−3.79	6.58 × 10^−08^
SAUSA300_1683	***aroA2***	Chorismate mutase/phospho-2-dehydro-3-deoxyheptonate aldolase	−3.65	8.52 × 10^−06^
SAUSA300_1499	*aroK*	Shikimate kinase	−3.55	1.09 × 10^−18^
SAUSA300_1555	*aroE*	Shikimate 5-dehydrogenase	−2.67	2.48 × 10^−15^
Isoprenoid biosynthesis				
SAUSA300_0355	***SAUSA300_0355***	Acetyl-CoA acetyltransferase	−8.15	1.26 × 10^−61^
SAUSA300_2484	*mvaS*	Hydroxymethylglutaryl-CoA synthase	−7.37	1.01 × 10^−54^
SAUSA300_2483	*mvaA*	Hydroxymethylglutaryl-CoA reductase	−7.16	4.96 × 10^−67^
Central carbon metabolism				
SAUSA300_1193	***glpD***	Glycerol-3-phosphate dehydrogenase	−10.35	4.97 × 10^−47^
SAUSA300_2455	***fbp***	Putative fructose-1,6-bisphosphatase	−7.40	1.27 × 10^−40^
SAUSA300_1916	*aspB*	Aminotransferase	−5.88	1.68 × 10^−125^
SAUSA300_1246	*acnA*	Aconitate hydratase	−3.36	1.12 × 10^−10^
SAUSA300_1640	*icd*	Isocitrate dehydrogenase, NADP-dependent	−2.65	3.44 × 10^−08^
SAUSA300_2362	*gpmA*	2,3-Bisphosphoglycerate-dependent phosphoglycerate mutase	−2.39	3.06 × 10-^21^
SAUSA300_1014	*pyc*	Pyruvate carboxylase	−2.08	5.08 × 10^−19^
SAUSA300_1190	*glpP*	Glycerol uptake operon antiterminator regulatory protein	−2.19	1.17 × 10^−16^
Nucleotide biosynthesis				
SAUSA300_1889	***purB***	Adenylosuccinate lyase	−6.16	1.62 × 10^−85^
SAUSA300_0017	***purA***	Adenylosuccinate synthetase	−5.58	4.28 × 10^−43^
SAUSA300_0965	*folD*	Methylenetetrahydrofolate dehydrogenase/methenyltetrahydrofolate cyclohydrolase	−4.46	6.25 × 10^−36^
SAUSA300_0473	*purR*	Pur operon repressor	−4.26	7.38 × 10^−06^
SAUSA300_2526	*pyrD*	Dihydroorotate dehydrogenase	−2.28	1.15 × 10^−13^
Transport and nutrient uptake systems				
SAUSA300_2406	*cntE*	Putative transporter	−9.72	0
SAUSA300_0620	*mntA*	ABC transporter ATP-binding protein	−7.60	6.93 × 10^−39^
SAUSA300_0619	*mntB*	ABC transporter, permease protein	−7.19	2.24 × 10^−26^
SAUSA300_0618	*mntC*	ABC transporter, substrate-binding protein	−6.60	3.90 × 10^−28^
SAUSA300_0633	*fhuA*	Ferrichrome transport ATP-binding protein fhuA	−6.05	1.60 × 10^−59^
SAUSA300_2306	*hrtA*	ABC transporter, ATP-binding protein	−3.67	3.92 × 10^−25^
SAUSA300_2307	*hrtB*	ABC transporter, permease protein	−3.55	7.51 × 10^−29^
SAUSA300_2308	***hssR***	Response regulator protein	−3.53	1.04 × 10^−44^
SAUSA300_1785	*ecsB*	Putative ABC transporter protein EcsB	−3.13	1.16 × 10^−30^
SAUSA300_2309	*hssS*	Sensor histidine kinase	−2.91	2.13 × 10^−26^
SAUSA300_1786	*ecsA*	ABC transporter, ATP-binding protein EcsA	−2.80	6.54 × 10^−14^
SAUSA300_1516	*znuC*	ABC transporter, ATP-binding protein	−2.30	3.73 × 10^−11^
SAUSA300_1515	*znuB*	ABC transporter, permease protein	−2.26	4.77 × 10^−15^
SAUSA300_0687	*mpfA*	Putative hemolysin	−2.06	4.21 × 10^−27^
Chromosome maintenance and cell division				
SAUSA300_1127	*smc*	Chromosome segregation protein SMC	−4.72	2.46 × 10^−27^
SAUSA300_1120	*recG*	ATP-dependent DNA helicase RecG	−3.54	1.54 × 10^−21^
SAUSA300_1086	*divIVA*	Putative cell-division initiation protein	−2.51	1.40 × 10^−12^
SAUSA300_1793	*SAUSA300_1793*	Conserved hypothetical protein	−2.35	5.25 × 10^−07^
SAUSA300_1792	*SAUSA300_1792*	Conserved hypothetical protein	−2.28	5.92 × 10-^07^
Cell envelope biogenesis and lipoprotein processing				
SAUSA300_0744	*lgt*	Prolipoprotein diacylglyceryl transferase	−3.44	5.68 × 10^−15^
SAUSA300_0980	*auxA*	Putative membrane protein	−3.36	1.52 × 10^−28^
SAUSA300_0918	*ugtP*	Diacylglycerol glucosyltransferase	−3.22	2.51 × 10^−11^
SAUSA300_2439	*galU*	UTP-glucose-1-phosphate uridylyltransferase	−2.96	6.65 × 10^−07^
SAUSA300_1089	*lspA*	Lipoprotein signal peptidase	−2.26	1.03 × 10^−09^
Virulence- and stress-related genes				
SAUSA300_2100	*SAUSA300_2100*	Lytic regulatory protein	−4.71	3.25 × 10^−60^
SAUSA300_2467	*srtA*	Sortase	−4.49	1.03 × 10^−16^
SAUSA300_2245	*sarR*	Staphylococcal accessory regulator R	−4.43	3.76 × 10^−22^
SAUSA300_2506	*isaA*	Immunodominant staphylococcal antigen A precursor	−4.40	9.25 × 10^−30^
SAUSA300_1842	*perR*	Transcriptional regulator, Fur family	−2.61	7.79 × 10^−10^
SAUSA300_1257	*msrR*	Peptide methionine sulfoxide reductase regulator MsrR	−2.34	1.75 × 10^−11^
SAUSA300_0310	*pfoR*	Perfringolysin O regulator protein	−2.33	1.79 × 10^−54^
SAUSA300_2249	*ssaA*	Secretory antigen precursor SsaA	−2.11	1.49 × 10^−13^
Two-component system				
SAUSA300_0022	*walH*	YycH protein	−3.03	5.05 × 10^−42^
SAUSA300_0023	*walI*	YycI protein	−2.78	7.33 × 10^−21^
Others				
SAUSA300_1899	***pncA***	Conserved hypothetical protein	−4.86	6.67 × 10^−43^
SAUSA300_0443	*SAUSA300_0443*	YibE/F-like protein	−4.64	1.33 × 10^−34^
SAUSA300_0442	*SAUSA300_0442*	YibE/F-like protein	−4.40	3.51 × 10^−27^
SAUSA300_1231	*SAUSA300_1231*	Gamma-aminobutyrate permease	−3.02	1.88 × 10^−31^
SAUSA300_1155	*rasP*	Putative membrane-associated zinc metalloprotease	−3.02	9.88 × 10^−19^
SAUSA300_2075	*rho*	Transcription termination factor Rho	−2.73	2.86 × 10^−08^
SAUSA300_1289	*dapB*	Dihydrodipicolinate reductase	−2.67	2.49 × 10^−14^
SAUSA300_1583	*cymR*	Conserved hypothetical protein	−2.60	1.69 × 10^−14^
SAUSA300_1017	*ctaM*	Conserved hypothetical protein	−2.56	1.47 × 10^−09^
SAUSA300_1112	*stp1*	Protein phosphatase 2C domain protein	−2.48	1.74 × 10^−15^
SAUSA300_0957	*SAUSA300_0957*	Conserved hypothetical protein	−2.42	5.99 × 10^−15^
SAUSA300_2156	*lacR*	Lactose phosphotransferase system repressor	−2.40	2.94 × 10^−06^
SAUSA300_2037	*cshA*	ATP-dependent RNA helicase	−2.24	4.09 × 10^−07^

^*a*^ Genes shown in bold were selected for further validation.

### Respiratory and carbon metabolic pathways support *S. aureus* fitness in human blood

**Fig 2:**
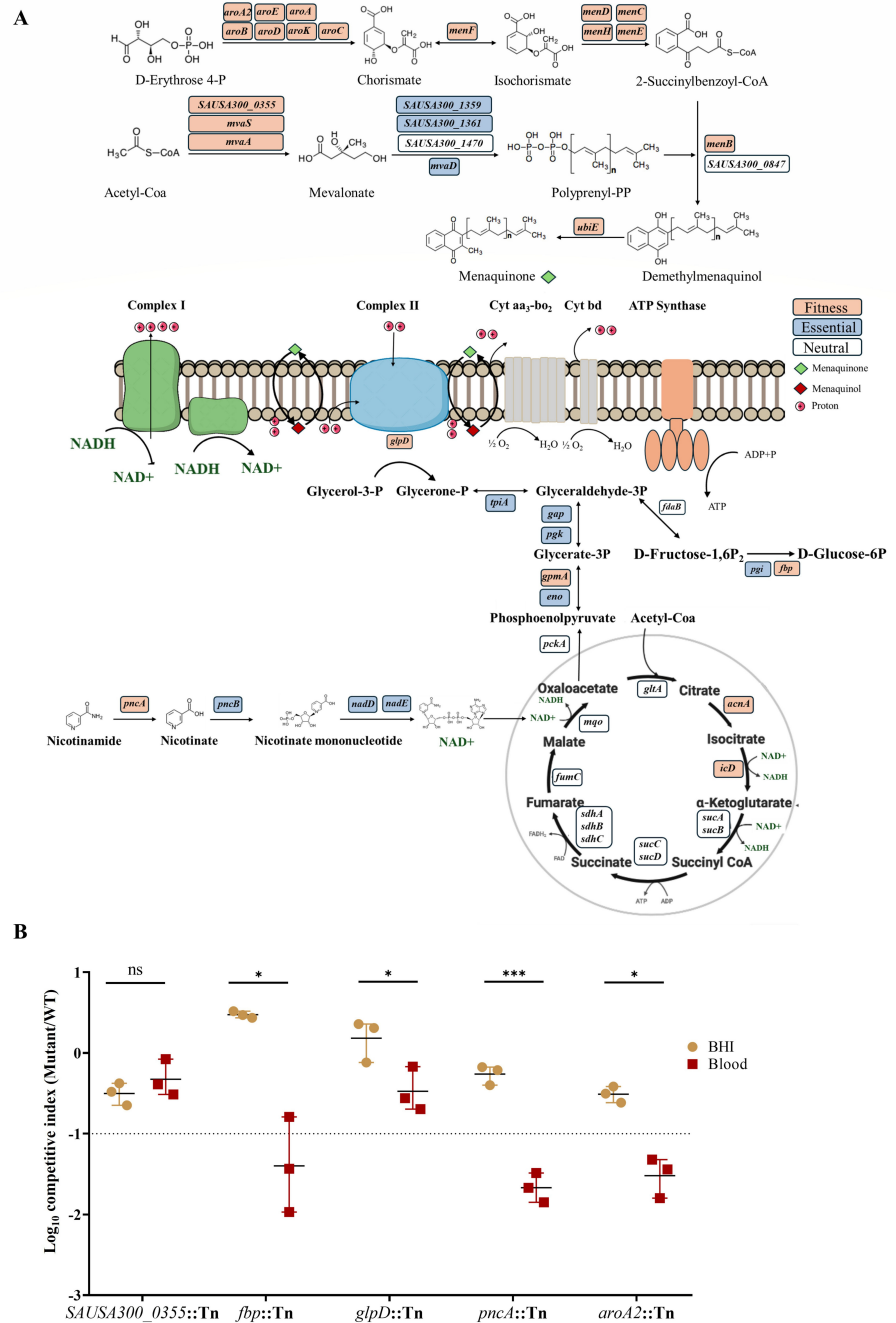
Schematic overview of fitness-associated gene depletion in the menaquinone pathway and central carbon metabolism of *S. aureus*. (**A**) The diagram illustrates the menaquinone biosynthesis pathway, gluconeogenesis, and the NAD^+^ salvage pathway, highlighting genes that contribute to bacterial fitness in human blood. Gene fitness was assessed using TraDIS analysis. Genes are color-coded based on fitness classification: orange indicates genes whose disruption significantly reduced fitness in blood (Log_2_FC ≤ −2, q-value < 0.01, logCPM > 2, mean control reads > 150), blue denotes genes essential for survival under standard laboratory conditions, and white represents genes with no significant fitness impact or lacking statistical significance. (**B**) Competitive assays in which *SAUSA300_0355, fbp, glpD, pncA*, and *aroA2* mutants were each co-cultured with WT during growth in BHI and human blood. Data represent mean ± SD of biological replicates. The dashed line represents the cut-off (log_10_CI = −1). Statistical significance was assessed using a two-tailed unpaired Welch’s *t*-test. Asterisks indicate adjusted *P* values: *P < 0.05, ***P < 0.001, ns = not significant.

TraDIS analysis revealed that *S. aureus* fitness in human blood depends on an intact electron transport chain (ETC) and energy metabolism pathways. Mutants disrupted in chorismate biosynthesis (*aroA, aroE*, *aroB*, *aroD*, *aroK*, *aroC*, *aroA2*) and isoprenoid biosynthesis (*SAUSA300_0355*, *mvaA*, *mvaS*) were significantly underrepresented following blood exposure. These pathways intersect with menaquinone biosynthesis (*menF, menD, menC, menH, menE, menB, menG, ubiE*), which also showed marked depletion in mutant representation (Table 1; Table S2A and B; Fig. 2A).

In addition to the menaquinone biosynthesis, *glpD* (glycerol-3-phosphate dehydrogenase) and its regulator *glpP* emerged as critical for *S. aureus* blood fitness. *glpD* links glycerol catabolism to respiratory electron transfer and gluconeogenesis (Fig. 2A). Transposon insertions in genes encoding gluconeogenic enzymes (*fbp, gpmA*) directing carbon flux from glycerone-P to glucose were underrepresented. Similarly, mutants in tricarboxylic acid (TCA) cycle genes (*acnA, icd*) and NAD^+^ biosynthesis gene *pncA* showed significantly reduced representation compared to outgrowth controls (Table 1; Table S2B).

To validate these findings, transposon mutants of *aroA2*, *SAUSA300_0355*, *glpD*, *pncA*, and *fbp* were subjected to fitness competition assays against the wild-type (WT) strain in BHI and human blood. Mutants lacking *aroA2*, *pncA*, or *fbp* exhibited pronounced fitness defects in blood relative to BHI (Fig. 2B). The *glpD* mutant was modestly reduced in human blood relative to BHI but stayed above the defect threshold (log_10_ competitive index [log_10_CI] < −1), while *SAUSA300_0355* mutants showed no significant competitive fitness defect in blood (Fig. 2B; Table S3A and B).

### Purine auxotrophy impairs *S. aureus* fitness during bloodstream infection

**Fig 3:**
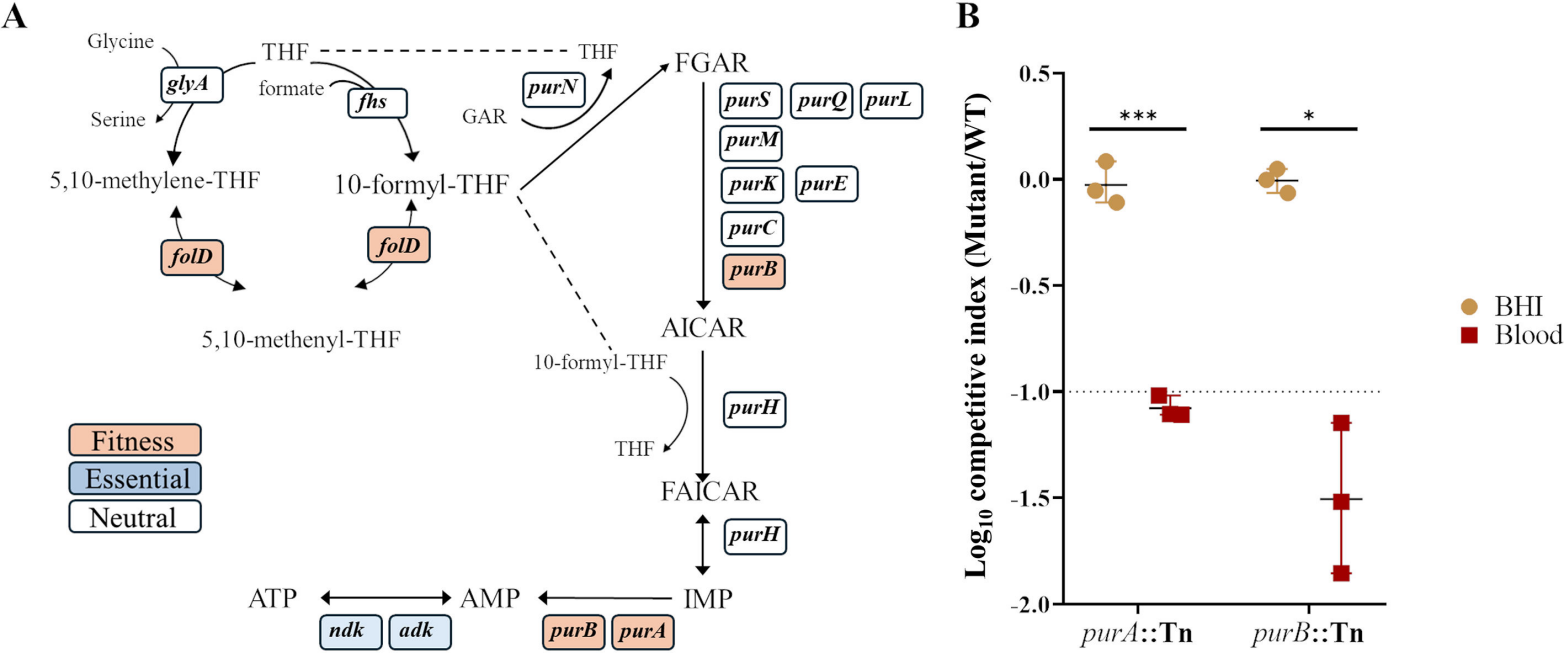
*De novo* purine biosynthesis is essential for *S. aureus* fitness in human blood. (**A**) Schematic representation of the *de novo* purine biosynthesis pathway. Genes are color-coded to indicate fitness, with orange for reduced fitness in blood, blue for essential genes under standard conditions, and white for genes without a significant effect. (**B**) Competitive assay of *purA* and *purB* transposon mutants relative to the WT strain during *ex vivo* survival in human blood. Individual data values correspond to biological replicates, shown as mean ± SD. The dashed line marks the cut-off log_10_CI equal to −1. Statistical significance was assessed using a two-tailed unpaired Welch’s *t*-test. Adjusted *P* values: *P < 0.05, ***P < 0.001.

The *de novo* purine biosynthesis pathway was identified as required for *S. aureus* fitness in human blood. Among the most significantly depleted mutants were *purA* (Log_2_FC = –5.58) and *purB* (Log_2_FC = –6.16) (Table 1; Table S2A and B; Fig. 3A). Additionally, *folD*, which encodes a bifunctional enzyme involved in one-carbon metabolism and purine precursor synthesis, also showed substantial depletion. To validate these findings, *ex vivo* competition assays demonstrated that *purA* and *purB* mutants were significantly outcompeted by the WT strain in blood (Fig. 3B; Table S3A and B).

### Maintenance of metal acquisition and heme detoxification systems enhances *S. aureus* fitness in blood

**Fig 4:**
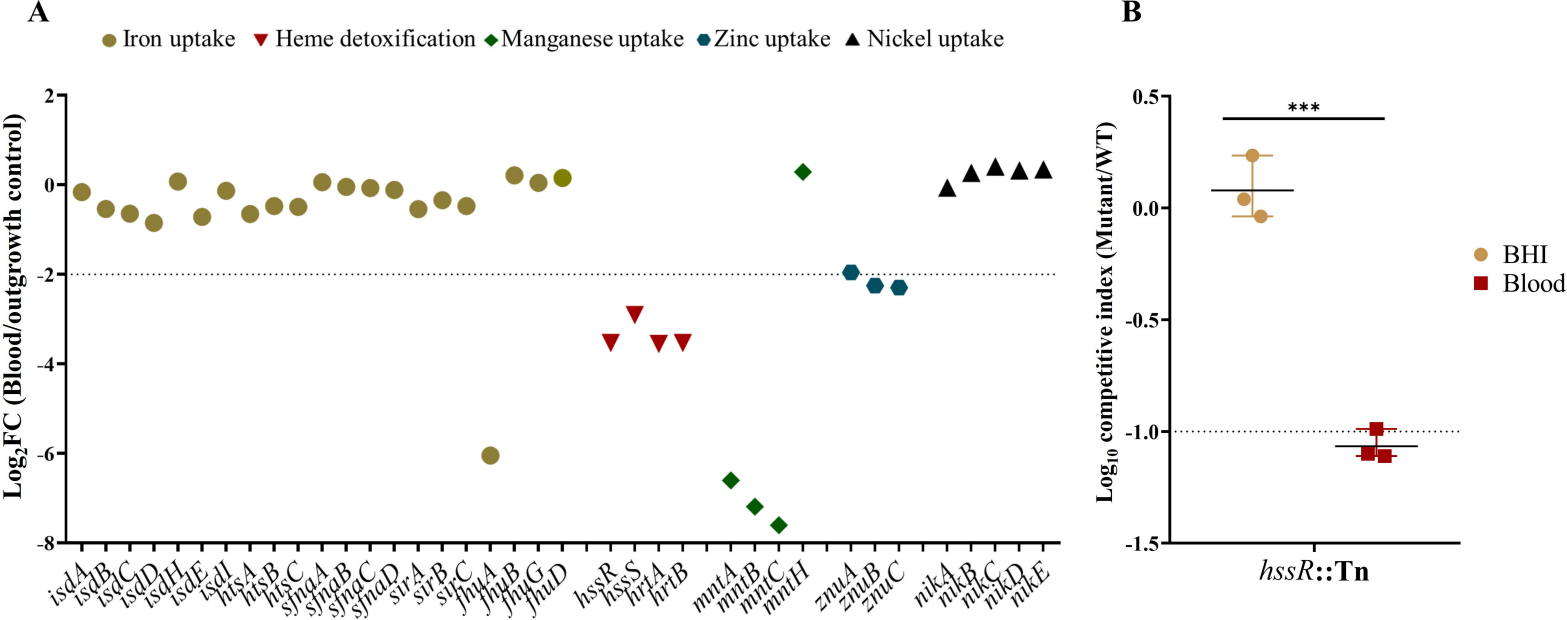
Heme detoxification and metal ion uptake systems are essential for *S. aureus* fitness in human blood. (**A**) Log_2_FC values from TraDIS analysis of *S. aureus* transposon mutants exposed to human blood. Genes included those involved in the heme detoxification system, manganese transporter, zinc transporter, nickel transporter, and iron uptake. Genes with Log_2_FC ≤ −2 (q-value < 0.01, logCPM > 2, mean control reads > 150) are considered as conditionally essential in human blood. (**B**) Competitive assay of the *hssR* mutant and WT strain during co-incubation in human blood. Biological replicates are shown as mean ± SD. The dashed line indicates the cut-off log_10_CI equal to −1. Statistical significance was assessed using a two-tailed unpaired Welch’s *t*-test. Significance levels are indicated as *** (*P* < 0.001).

We also identified several genes involved in metal ion acquisition and heme detoxification that contribute to *S. aureus* fitness in human blood. Among iron uptake systems, only *fhuA*, encoding a ferrichrome-specific ATP-binding cassette (ABC) transporter, was significantly depleted following blood exposure. Similarly, transposon insertions in manganese transport genes (*mntA, mntB, mntC*) and zinc transport components (*znuB, znuC*) were markedly underrepresented (Fig. 4A).

Disruption of the heme detoxification machinery, comprising the *hssRS* heme-sensing two-component system and the associated *hrtAB* efflux transporter, also resulted in a significant loss of fitness in blood (Fig. 4A). To experimentally assess this effect, an *hssR* transposon mutant was tested in competition assays, exhibiting reduced competitiveness in human blood relative to BHI (Fig. 4B; Table S3A and B).

### Loss of the Sae and σ^B^ regulatory systems confers a selective advantage for *S. aureus* bloodstream fitness

**Fig 5:**
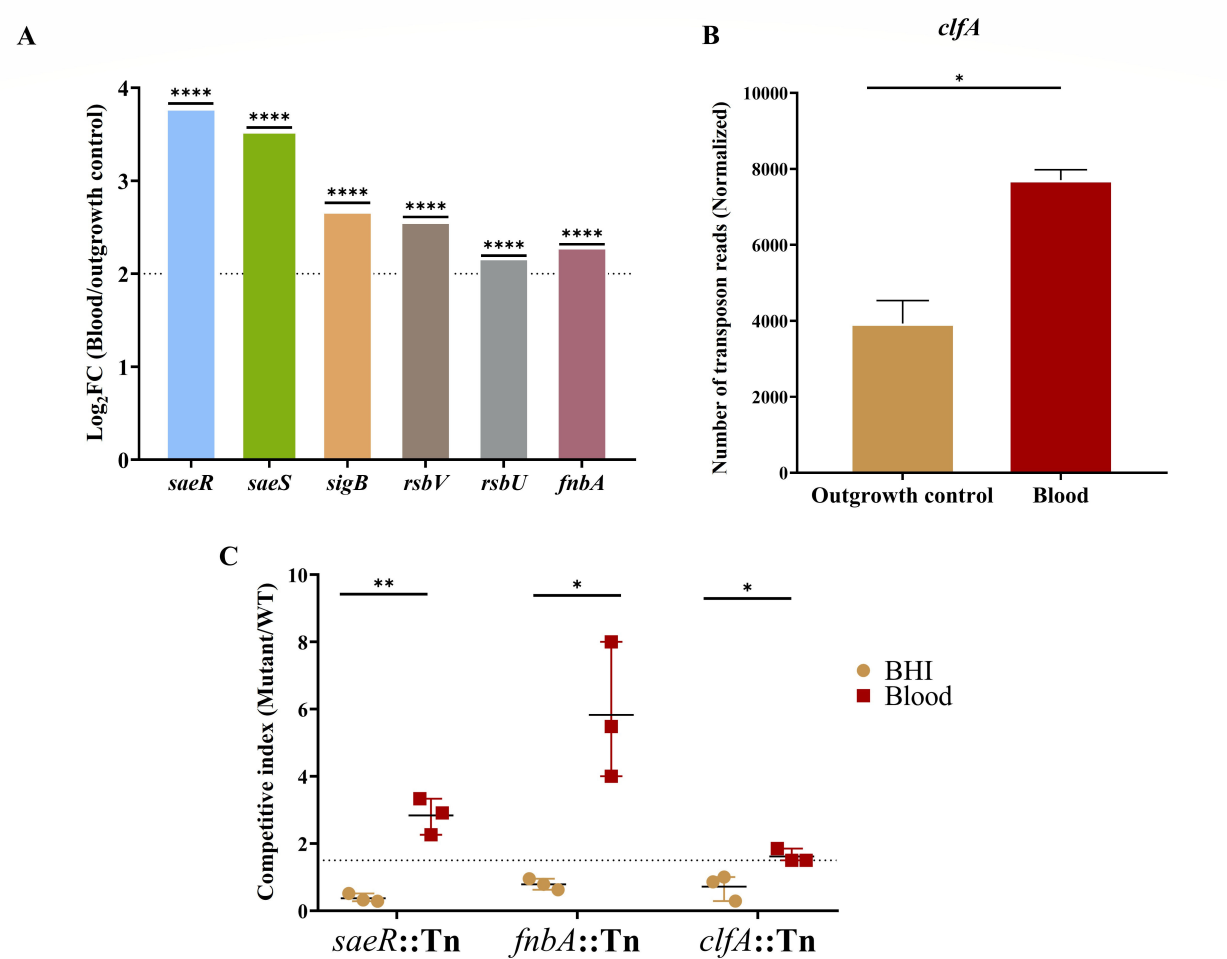
Inactivation of regulatory and adhesion genes enhances *S. aureus* fitness in human blood. (**A**) Log_2_FC values from TraDIS analysis for genes whose inactivation conferred a selective advantage during survival in human blood (Log_2_FC > 2, q-value < 0.01, logCPM > 2, mean control reads > 150). Statistical significance is indicated by asterisks, with **** corresponding to q-value < 0.0001. All q-values are reported in Table S1B. (**B**) Normalized transposon read counts for *clfA* are shown as mean ± SD of sequencing replicates. Statistical significance was determined by BioTraDIS analysis (Table S2B), with * indicating q-value = 0.016. (**C**) Fitness competition assays of mutants relative to WT during 1:1 co-culture in human blood. Data are shown as mean ± SD of three independent biological replicates. Statistical significance was assessed using a two-tailed unpaired Welch’s *t*-test. Adjusted *P* values: *P < 0.05, **P < 0.01.

TraDIS analysis revealed six mutants significantly enriched following exposure to human blood (Table S2A and B). These included disruptions of the *saeRS* two-component system and genes of the alternative sigma factor σ^B^ operon (*sigB*, *rsbV*, and *rsbU),* which were among the most strongly enriched (Fig. 5A). Notably, mutants in *fnbA*, co-regulated by both the Sae and σ^B^ systems, were also highly enriched. Although *clfA* did not reach the statistical enrichment threshold, it displayed a modest increase in transposon read abundance and was retained for validation due to its known σ^B^ regulation alongside *fnbA* and *saeR* mutants (Fig. 5B). All selected enriched mutants showed a significant fitness advantage over WT in competitive assays within the human blood environment (Fig. 5C; Table S3A and B). To ensure that the enrichment represented a biologically meaningful advantage, we applied a cutoff of log₁₀CI > 0.17 (CI ≥ 1.5), corresponding to a ≥50% increase in fitness, which is more stringent than the log₁₀CI > 0 (CI ≥ 1) used in previous studies (18, 19).

### Fitness landscape between community- and livestock-associated MRSA

**Fig 6:**
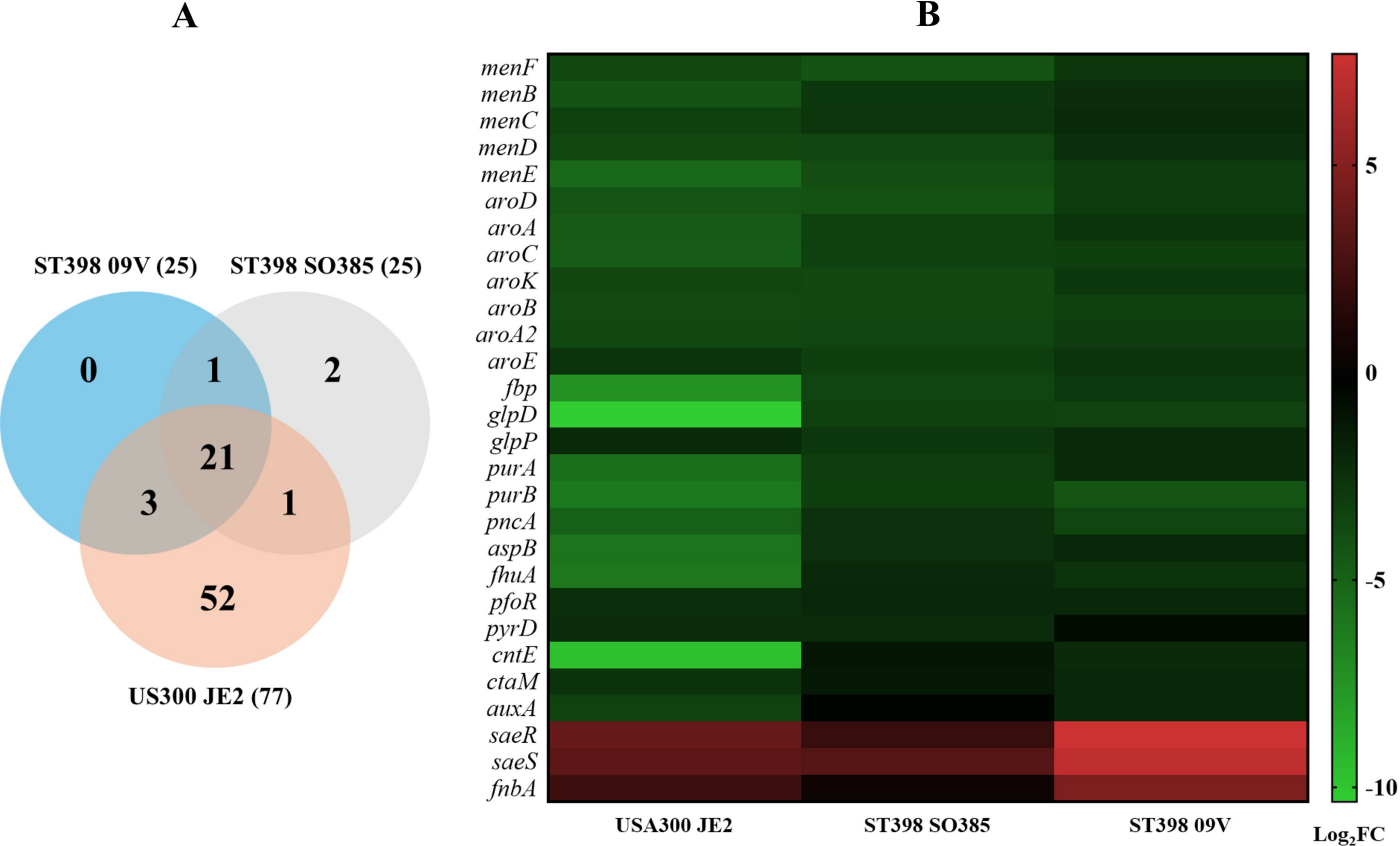
Comparative TraDIS analysis identifies conserved fitness determinants in *S. aureus*. (**A**) Venn diagram showing the overlap of fitness-associated genes identified via TraDIS in *S. aureus* USA300 JE2, ST398 SO385, and ST398 09V strains during survival in human blood. (**B**) Heatmap showing Log₂FC values of transposon mutant abundance conserved between USA300 JE2 and at least one ST398 strain, highlighting both fitness-enhancing (green) and fitness-reducing (red) loci.

To identify conserved and lineage-specific fitness determinants in human blood, we compared our TraDIS dataset from the CA-MRSA strain USA300 JE2 with two publicly available datasets from LA-MRSA strains SO385 and 09V grown in human blood (12). Both strains belong to Sequence Type 398 (ST398) within Clonal Complex 398 (CC398), allowing for a direct comparison of fitness requirements between divergent lineages in the blood environment. We identified 21 genes consistently required for blood fitness across all three strains (Log_2_FC ≤ –2, q-value < 0.01, logCPM > 2, mean control reads > 150), primarily involved in menaquinone biosynthesis and purine metabolism (Fig. 6A and B). In addition, we observed consistent enrichment of transposon reads of the *saeRS* and *fnbA* genes in all three strains, where components of the σ^B^ operon were selectively enriched in USA300 JE2 (Fig. 6B).

## DISCUSSION

To establish infection in the bloodstream, *S. aureus* must adapt to multiple host-imposed stresses, including nutrient limitation, oxidative burst, and immune recognition (20). These challenges drive extensive metabolic reprogramming and coordinated expression of virulence factors that enable persistence across diverse host environments (20, 21). Several high-throughput genetic approaches have been applied to investigate fitness requirements during bloodstream infection. For example, phenotypic screens utilizing the Nebraska transposon mutant library (NTML) have identified mutants with impaired growth on blood agar plates (22). Additionally, genome-wide TraDIS analyses of livestock-associated MRSA lineages, including ST398, have revealed genes that contribute to fitness in human blood (12). Chang et al. recently performed a Tn-seq screen of USA300 in commercially processed, leukocyte-depleted blood (15). While these studies offered important insights, a genome-wide analysis of CA-MRSA fitness in fresh, immune-competent human blood has been lacking.

To address this gap, we conducted a genome-wide TraDIS screen using a highly saturated transposon mutant library constructed in the USA300 JE2 strain to systematically identify the genetic determinants essential for fitness in human blood (16). A major drawback of transposon mutant sequencing studies is the bottleneck effect, in which only a fraction of the initial mutant pool contributes to the surviving population, reducing diversity and compromising statistical power (23). To mitigate this limitation, a high inoculum was used to preserve library representation, consistent with strategies validated in infection models (24, 25). Despite the enrichment of mutants with specific fitness advantages under blood-specific pressures, overall library richness and gene representation were found to be preserved. Thus, the bottleneck observed in our TraDIS screen using fresh human blood was selective rather than restrictive and did not compromise downstream analyses. Preservation of the transposon library diversity is fundamental, as fresh blood represents a complex milieu of viable immune cells, active complement, antimicrobial peptides, and nutritional immunity absent in processed preparations (4, 26). The high biological relevance of this environment, coupled with preserved library complexity, enabled robust identification of clinically important genetic traits involved in fitness in human blood. Despite its recent decline in prevalence, USA300 remains a clinically significant CA-MRSA lineage, and its well-annotated genome provides a robust platform for functional genomics (27). Our screen identified 76 fitness-associated genes, and six genes whose disruption enhanced fitness in human blood. Although bacterial fitness in blood does not capture the full complexity of *in vivo* pathogenesis, it represents a crucial early step in invasive disease (28). Several genes identified in our screen, including *pyc*, *SAUSA300_1231*, *lgt*, *lspA*, *msrR*, *srtA*, *gpmA*, *mntABC*, *cntE*, *stp1, cymR,* and *isaA*, have previously been validated in animal models of systemic infection or proposed as immunotherapeutic targets, highlighting the biological relevance of our findings (15, 29–36).

Our TraDIS screen highlighted *de novo* purine biosynthesis pathway as a critical determinant of *S. aureus* fitness during bloodstream infection, evidenced by the significant depletion of *purA* and *purB*. This pathway is essential for sustaining intracellular pools of purine nucleotides necessary for DNA replication, RNA transcription, and energy metabolism (37). While *S. aureus* can salvage exogenous purines under nutrient-rich conditions, the human bloodstream represents a nutrient-restricted environment, where purine availability is constrained by host-mediated sequestration mechanisms and rapid turnover of circulating nucleotides (38). While previous studies have reported inconsistent validation outcomes in blood (12, 15, 22), our competitive assays confirmed that the *purA* and *purB* mutants are significantly impaired in blood, affirming the necessity of metabolic autonomy in nucleotide biosynthesis for bloodstream fitness (15, 22). Given their essentiality in multiple infection models, including murine pneumonia and osteomyelitis, purine biosynthetic enzymes have been proposed as attractive antimicrobial targets (7, 29). However, their considerable structural homology to mammalian counterparts poses a major challenge in developing selective inhibitors.

Respiratory metabolism is a cornerstone of *S. aureus* physiology, supporting ATP generation, redox homeostasis, and metabolic flexibility (39). In line with its central role in bacterial fitness, our genome-wide TraDIS screen in blood revealed a pronounced depletion of transposon mutants in genes encoding key components of ETC, including those required for the biosynthesis of chorismate (*aroA, aroE*, *aroB*, *aroD*, *aroK*, *aroC*, *aroA2*), mevalonate (*SAUSA300_0355*, *mvaA*, *mvaS*), and menaquinone (*menF, menD, menC, menH, menE, menB, menG, ubiE*). Menaquinone serves as the predominant quinone electron carrier in *S. aureus*, and its biosynthetic disruption impairs aerobic respiration, resulting in profound metabolic consequences (39, 40). Such defects often lead to small colony variants, a subpopulation with reduced growth, diminished virulence, and enhanced intracellular persistence, traits that confer a short-term survival advantage after exposure to blood (39, 41). However, our competitive fitness assays revealed that mutants, such as *aroA2,* showed reduced long-term fitness in blood, suggesting that transient stress tolerance does not substitute for the robust metabolic capacity required to sustain adaptation and persistence in this nutrient-limited environment. In accordance with these findings, our previous genome-wide TraDIS screens in murine infection models revealed that genes in the mevalonate, menaquinone, and chorismate pathways were differentially required depending on the host niche (24). In a systemic infection model, mutants disrupted in *SAUSA300_0355*, *mvaA*, *mvaS*, *menE*, *aroB*, and *aroC* were depleted in transposon libraries recovered from the kidney, whereas in a skin infection model, mutants disrupted in *menF* and *ubiE* were enriched (24). These data demonstrate how host niche-specific conditions shape metabolic fitness requirements and highlight the importance of interpreting genome-wide fitness screens within their environmental context.

*S. aureus* reprograms its carbon metabolism in human blood in response to the available nutrients rather than following the hierarchical substrate preferences observed in rich laboratory media (15). Human blood provides only modest glucose, together with lactate and free amino acids, and *S. aureus* can assimilate these substrates concurrently rather than sequentially (15). Consistent with this metabolic flexibility, our genome-wide TraDIS screen identified a set of genes that channel diverse carbon sources into central metabolism and biosynthesis, including *fbp* and *gpmA*, anaplerotic enzymes such as *pyc* and *aspA*, and glycerol catabolic functions *glpD* and *glpP*, as key determinants of fitness. *fbp*, which encodes fructose-1,6-bisphosphatase that produces hexose phosphates to feed the pentose phosphate pathway and support anabolic processes, was selected for competition assays and exhibited a pronounced fitness defect, confirming its broad requirement in blood consistent with tissue-specific studies (15, 24). In contrast, other gluconeogenic nodes, including *pckA* and *gapB*, did not emerge as fitness determinants, consistent with previous reports suggesting context dependence (24). Although staphylococcal infections have often been described as glycolysis dependent, *pyk*, encoding pyruvate kinase and previously identified as essential in osteomyelitis, was not required for fitness in our human blood screen (42, 43). This observation aligns with prior studies in which *pyk* similarly did not contribute to fitness across diverse infection models (15, 24).

Mutants in TCA cycle enzymes, including *acnA* and *icd*, were also depleted, highlighting the central role of the TCA cycle in generating reducing equivalents for the ETC and supporting oxidative-stress defense (44). These results demonstrate the metabolic plasticity of *S. aureus*, which allows the pathogen to dynamically recalibrate its central pathways in response to the diverse host environments encountered during infection.

Importantly, this integration of central carbon metabolism and TCA cycle activity depends on NAD^+^ availability. PncA sustains NAD^+^ pools by converting nicotinamide to nicotinic acid, fueling NAD^+^ regeneration and allowing continuous TCA flux (45). This, in turn, ensures a steady supply of NADH to the electron transport chain, supporting ATP production and maintaining redox homeostasis (46). In blood, transposon insertions in *pncA* were significantly depleted, a finding that was further confirmed by phenotypic assays. Notably, PncA was recently identified as a novel regulator of the Agr system, and its loss was shown to reduce virulence in both *Galleria mellonella* and murine infection models (47). Together, these findings support a model in which *S. aureus* fitness in blood relies on an integrated energy hub that couples glycerol and amino acid catabolism, gluconeogenesis, TCA-cycle flux, and NAD^+^ regeneration to sustain respiratory ATP production, redox balance, and resistance to oxidative stress.

The bloodstream imposes additional constraints on pathogens via nutritional immunity, limiting access to essential metals, such as iron, manganese, and zinc (48). Consistent with this, TraDIS results showed that mutants in manganese (*mntA, mntB, mntC*) and zinc (*znuB, znuC*) acquisition systems were found to contribute to bacterial fitness in blood. While iron acquisition pathways are known to be upregulated during infection, our screen revealed only *fhuA*, encoding an ATPase essential for siderophore-mediated iron uptake, as a fitness determinant in blood (10). FhuA supports the import of staphyloferrin A and B, as well as hydroxamate-type siderophores, likely explaining its non-redundant function in iron acquisition under iron-restricted conditions (49).

During bloodstream infection, hemoglobin-derived heme serves as a primary iron source for *S. aureus*. However, while heme is a rich reservoir of iron, its intracellular accumulation poses a significant risk for bacterial fitness due to its pro-oxidant potential. Free heme can drive the production of reactive oxygen species that inflict oxidative damage on cellular macromolecules, including lipids, proteins, and nucleic acids (50). Our TraDIS screen identified the *hssRS* two-component regulatory system and its associated efflux transporter *hrtAB* as key contributors to bacterial fitness in blood. *hssRS* functions as a heme-responsive sensor that activates *hrtAB*-mediated efflux in response to toxic intracellular heme levels. This was validated by the reduced competitiveness of *hssR* transposon mutant. Previous *in vivo* studies further demonstrated that disruption of *hssRS* or *hrtAB* impairs USA300 fitness in the spleen but not in the kidney, lung, and liver (15, 24). This tissue-specific requirement likely reflects the spleen’s function as a primary site of erythrocyte turnover and hemoglobin catabolism, which creates a locally concentrated, heme-rich environment (51). However, disruption of *hssRS* or *hrtAB* in the ST398 lineage did not affect fitness in either human or porcine blood, suggesting lineage-specific differences in heme handling or compensatory detoxification mechanisms, pointing to a broader diversity in metal homeostasis strategies across *S. aureus* strains (12).

To assess conservation of fitness determinants, we reanalyzed TraDIS data from two MRSA ST398 strains (12). We found that 21 fitness genes overlapped, including genes involved in menaquinone and purine biosynthesis. For some genes, we observed differences between lineages. These differences may reflect lineage-specific gene regulation or host adaptation, beyond technical variability.

Interestingly, we also identified several genes whose inactivation enhanced fitness in human blood. Insertions in *saeRS* and the σ^B^ operon (*sigB*, *rsbU*, *rsbV*).

These regulators control a broad array of surface-associated proteins, secreted factors, and stress response pathways (52, 53). SaeRS is essential for cytotoxicity and intracellular survival in mammalian host cells and has been reported to contribute to virulence in systemic infection models (15, 54). Paradoxically, loss of *saeRS* has been reported to increase survival in both human and murine blood (53, 54). Consistently, *saeRS* and *fnbA* were enriched when analyzing fitness-disadvantageous genes in livestock-associated MRSA strains exposed to human blood and in a USA300 transposon library challenged in immune-depleted blood, highlighting a context-dependent effect (12, 15). In this environment, where adhesive substrates are limited, expression of SaeRS- and σ^B^-regulated surface proteins, such as FnbA and ClfA, may impose a fitness cost. These data must be interpreted with nuance, as reduced expression of these genes may confer a fitness advantage in blood while they remain essential for tissue colonization, abscess formation, and systemic dissemination in *vivo* (55, 56).

To assess the influence of experimental context, we compared our results with a recent Tn-seq study in USA300 performed in commercially processed, leukocyte-depleted blood (15). Only 13 genes overlapped between the two datasets (*fhuA, purA, purB, srtA, glpD, fbp, mntA, mntB, mntC, cntE, pncA, ubiE, perR*) (15). These loci encode core metabolic and nutrient-acquisition functions that appear essential in blood irrespective of the presence of immune effectors. The limited overlap likely reflects both technical and biological factors. Technical differences may arise from variations in transposon library saturation and size, experimental design, and statistical thresholds. Biologically, our study employed fresh whole blood, which retains active immune components, including leukocytes, complement, antimicrobial peptides, and nutrient sequestration mechanisms (5). In contrast, Chang et al. used processed blood, in which immune cells are removed, and complement activity is largely inactivated (15). This physiological distinction likely underlies the divergent signatures observed, particularly within stress response and energy metabolism pathways. In processed blood, mutants lacking oxidative stress protection (*ahpC, ahpF*) and mutants in several DNA repair genes (*uvrA, uvrB, uvrC, recA, recG, recN, xseA, xseB, nfo, nth, polA*) were strongly enriched (15). This pattern reflects the reduced requirement for these defensive systems in the absence of leukocyte-derived reactive oxygen species and genotoxic pressure (57). Pathways that support oxidative stress defenses displayed an opposing fitness profile between whole blood and processed blood. Mutants in genes of the mevalonate pathway (*mvaA, mvaS*), which produce isoprenoid intermediates serving as precursors for menaquinone and the antioxidant carotenoid staphyloxanthin, were enriched in processed blood but depleted in whole blood (58). The same trend was observed for *acnA*, which encodes aconitase and links the tricarboxylic acid cycle to NADPH production (44). A recent study has shown that loss of *acnA* markedly reduces NADPH generation and staphyloxanthin levels, compromising the cell’s ability to detoxify reactive oxygen species (44). In the absence of immune cells and oxidative stress, these pathways become dispensable, and disrupting them appears to confer a fitness advantage. These observations illustrate that bacterial fitness is context-dependent and that the physiological environment can reshape the definition of fitness requirements.

Despite the successful identification and validation of several fitness determinants, we observed that some mutants depleted in TraDIS did not show fitness defects when tested individually. Such discrepancies are common in pooled mutant screens and may result from stochastic loss, sampling noise, or trans-complementation effects within complex transposon libraries (12, 23, 59, 60). Furthermore, the use of stringent thresholds in both TraDIS analysis and competitive index assays may exclude genes with moderate but biologically relevant effects. For example, *rpoE* (Log_2_FC = –1.8) has been previously shown to support fitness in blood (61). In competition assays, the choice of cut-off can affect classification. Previous studies considered mutants with log_10_CI < 0 as having reduced fitness relative to the WT, whereas we applied a more stringent threshold of log_10_CI < −1 (18, 19). This approach may explain why some mutants, such as *glpD*, were not identified as defective according to our criteria. In addition, although the high density of independent transposon insertions and concordant competitive assays provides internal validation, individual genetic complementation was not performed. While the NTML is a well-characterized resource, the potential for secondary mutations cannot be fully excluded. Finally, our *ex vivo* model relies on blood from healthy donors. Although this system is invaluable for controlled experimentation, it may not fully capture the complex immunometabolic landscape present during clinical bacteremia (62).

In summary, we identified and validated a set of genes that either promote or impair *S. aureus* fitness in human blood. These findings expand our understanding of CA-MRSA pathophysiology and demonstrate the utility of genome-wide fitness profiling in clinically relevant conditions. Future studies integrating *in vivo* validation and cross-lineage comparisons will enhance our knowledge of *S. aureus* pathogenesis and support the development of more effective targeted therapies.

## MATERIALS AND METHODS

### Bacterial strains and antibiotics

The strains used in this study are listed in Table S4. Transposon mutants were obtained from the NTML (63). Strains were cultured in BHI broth or on brain heart infusion agar (BHIA) (Microbiol, Cagliari, Italy). When required, erythromycin (5 mg/L) (Sigma-Aldrich, St. Louis, MO, USA) was added to the medium to select for transposon mutants.

### Screening of transposon mutant library in whole blood

Aliquots of the JE2 transposon mutant library were pooled, washed, and inoculated at approximately 4 × 10⁹ CFU into 10 mL of whole human blood obtained from three healthy volunteers. Blood was collected in lithium heparin vacutainer tubes (Hebei Xinle Sci&Tech Co., Ltd., Hebei, China) and used immediately upon collection. The inoculated blood cultures were processed independently and incubated at 37°C with aeration for 24 h. After the first incubation, CFU counts were determined on BHIA supplemented with erythromycin (5 mg/L). To enhance selection sensitivity and increase the bacteria-to-blood ratio, 100 µL from each blood culture was transferred into 9.9 mL of BHI broth and incubated at 37°C with aeration for an additional 24 h (12). Following the second growth round, genomic DNA was extracted from approximately 10^9^ mutants using the DNeasy Blood & Tissue Kit (Qiagen, Hilden, Germany) with the addition of 0.5 mg/mL (250 units/mL) of lysostaphin (Sigma Aldrich). The extracted DNA was considered as output and stored at −80°C for subsequent analysis. A preliminary survival assay in whole human blood was conducted to determine the optimal inoculum size. This step ensured that sufficient copies of each mutant were present to account for the expected initial decline in bacterial cell counts upon exposure to whole blood.

### TraDIS sequencing

Two micrograms of extracted DNA from the output libraries were resuspended in 100 μL of nuclease-free water and mechanically sheared to approximately 300 bp using the Bioruptor Pico sonicator (Diagenode, Liège, Belgium). The efficacy of the mechanical fragmentation process was assessed by evaluating the size distribution of the DNA fragments with a High-Sensitivity DNA Kit via the Agilent 2100 Bioanalyzer System (Agilent Technologies, Santa Clara, CA, USA). The fragmented DNA was subsequently processed to generate blunt-ended fragments using the NEBNext End Repair Module (New England Biolabs, Ipswich, MA, USA), followed by dA-tailing with the NEBNext dA-Tailing Module (New England Biolabs). Subsequently, the dA-tailed DNA was ligated to SpIA5 adapters using the NEBNext Quick Ligation Module. To enrich for transposon-inserted fragments, PCR amplification was performed using transposon-specific primers and adapter primers (Table S5) (13, 64). After each step of library preparation, DNA fragments were purified and size-selected using Agencourt AMPure XP beads (Beckman Coulter, Brea, CA, USA). The prepared libraries were sequenced on an Illumina MiSeq platform with the MiSeq V2 50-cycle reagent kit (Illumina), as previously described (64).

### TraDIS data analysis

Sequence reads obtained from both the input and output libraries were filtered, mapped, and analyzed using the BioTraDIS pipeline as modified by Garcia et al. (59, 64). Sequencing quality control of raw data was verified by FastQC and then processed using the *fq2bam.pl* script, which adds the transposon tag (TAAGAGACAG) at the end of the read name and converts the FASTQ files to SAM format. To add tags to the read strings, SAM files were converted to BAM files using Samtools 1.6, and then back to FASTQ files for further analysis. Using the Bacteria_tradis script, tags were filtered and removed from the resulting reads. These reads were then aligned to the reference genome (GenBank accession number: CP000255.1) using SMALT 0.7.6, with no mismatches permitted. As a result, unique transposon insertion sites and the corresponding read counts were identified for each gene. The *tradis_comparisons.R* script was used to assess the statistical significance of insertion differences between input and output data and to calculate Log_2_FC, after normalizing read counts with the trimmed mean of the M method in R. Significant genes (q-value < 0.01 and logCPM > 2) were categorized based on their transposon insertion profiles following exposure to human blood. Genes with Log_2_FC of ≤ −2 were classified as fitness genes, indicating depletion of transposon insertions under these conditions. Conversely, genes with a Log_2_FC > 2 were considered enriched, reflecting an increased representation of transposon insertions. To minimize false identification of fitness genes, we excluded genes with a mean read count below 150 in the outgrowth control (65). The selected genes were re-annotated using the AureoWiki database (http://aureowiki.med.uni-greifswald.de) and functionally characterized using the eggNOG-mapper 2.1.9.

### Diversity and bottleneck assessment

Insertional richness and diversity of the transposon mutant library during the *ex vivo* human blood assay were quantified at the gene level using read counts from input and output samples. Read counts were adjusted to account for differences in library size, and for each gene, proportional abundance (*p*_i_) was calculated as the gene’s count divided by the total counts across all genes. Shannon’s diversity index, Simpson’s diversity index, and Pielou’s evenness were computed from these proportional abundances using the vegan R package (v4.3.2). Rank abundance curves were generated by plotting the logarithm of proportional abundances against the ranked mutant lineages, allowing a visual comparison between input and output libraries. To further quantify potential bottlenecks, the effective population size (*N*_e_ₑ) was computed from proportional abundance data using the inverse Simpson diversity index:


$$N_{e}=\frac{1}{\sum p_{i}^{2}}$$

This metric reflects the number of equally abundant contributors effectively represented in the population (66). A decrease in the output relative to input indicates a reduction in library complexity in response to the selective pressures of the blood assay and is interpreted as evidence of a genetic bottleneck (67). PCA was performed on normalized insertion counts for all genes using the *factoextra* package to assess replicate similarity across input, outgrowth control, and blood conditions.

### *In vitro* competition assays

Overnight cultures of WT and mutant strains were washed and mixed at a 1:1 ratio, then inoculated into 5 mL of human blood in triplicate to achieve an initial inoculum of approximately 10⁷ CFU/mL. Cultures were incubated at 37°C for 24 h. After incubation, mutant counts were determined on erythromycin-supplemented plates. WT counts were calculated by subtracting mutant CFUs from the total CFUs obtained on plates without erythromycin. For assays conducted in BHI, the initial inoculum (T0) was used as the reference for CI calculations. However, preliminary experiments in blood revealed early population bottlenecks and rapid fluctuations in the mutant-to-WT ratio, which could bias measurements. Therefore, the bacterial population after 2 h of incubation (T1) was used as the reference inoculum. CI was calculated as the ratio of the mutant-to-WT population at the end of the assay divided by the mutant-to-WT ratio in the initial inoculum.

## Data Availability

Raw sequencing data and metadata are available in the NCBI Sequence Read Archive (SRA), under BioProject accession no. PRJNA1345259.

## SUPPLEMENTAL MATERIAL

Supplemental material spectrum.03585-25-s0001.docx Fig. S1 and S2; Tables S1, S4, and S5.

Table S2 spectrum.03585-25-s0002.xlsx tradis_comparison.R output for blood vs input and output for blood vs outgrowth control.

Table S3 spectrum.03585-25-s0003.xlsx Competitive index CFU counts in blood and in BHI medium.

ASM does not own the copyrights to Supplemental Material that may be linked to, or accessed through, an article. The authors have granted ASM a non-exclusive, world-wide license to publish the Supplemental Material files. Please contact the corresponding author directly for reuse.
