# Supplementary material for: Profiling of genetic determinants required for fitness of community-associated methicillin-resistant Staphylococcus aureus in human blood
Source: Microbiol Spectr. 2026 Apr 21;14(6):e03585-25. doi: 10.1128/spectrum.03585-25 (PMC13227979; doi:10.1128/spectrum.03585-25)
Supplement: Supplemental material — Fig. S1 and S2; Tables S1, S4, and S5. [file spectrum.03585-25-s0001.docx]

| Library | Library size | Total Reads | Mapped (%)^1^ | UIS^2^ | Total sequence length/ Total UIS^3^ |
| --- | --- | --- | --- | --- | --- |
| Input (Replicate 1)^4^ | 2.7 × 10^9^ | 14,654,230 | 78.39 | 315,400 | 9.10 |
| Input (Replicate 2)^4^ | 2.7 × 10^9^ | 10,210,994 | 79.6 | 392,797 | 7.31 |
| BHI (Replicate 1)^5^ | 1.2 × 10^9^ | 6,788,911 | 84.32 | 466,380 | 6.15 |
| BHI (Replicate 2)^5^ | 1.1 × 10^9^ | 8,167,660 | 94.14 | 429,496 | 6.68 |
| Blood (Replicate 1) | 2.1 × 10^9^ | 7,772,786 | 87.13 | 413,695 | 6.94 |
| Blood (Replicate 2) | 1.7 × 10^9^ | 8,221,349 | 87.47 | 402,077 | 7.14 |
| Blood (Replicate 3) | 1.2 × 10^9^ | 6,432,268 | 88.25 | 390,652 | 7.35 |


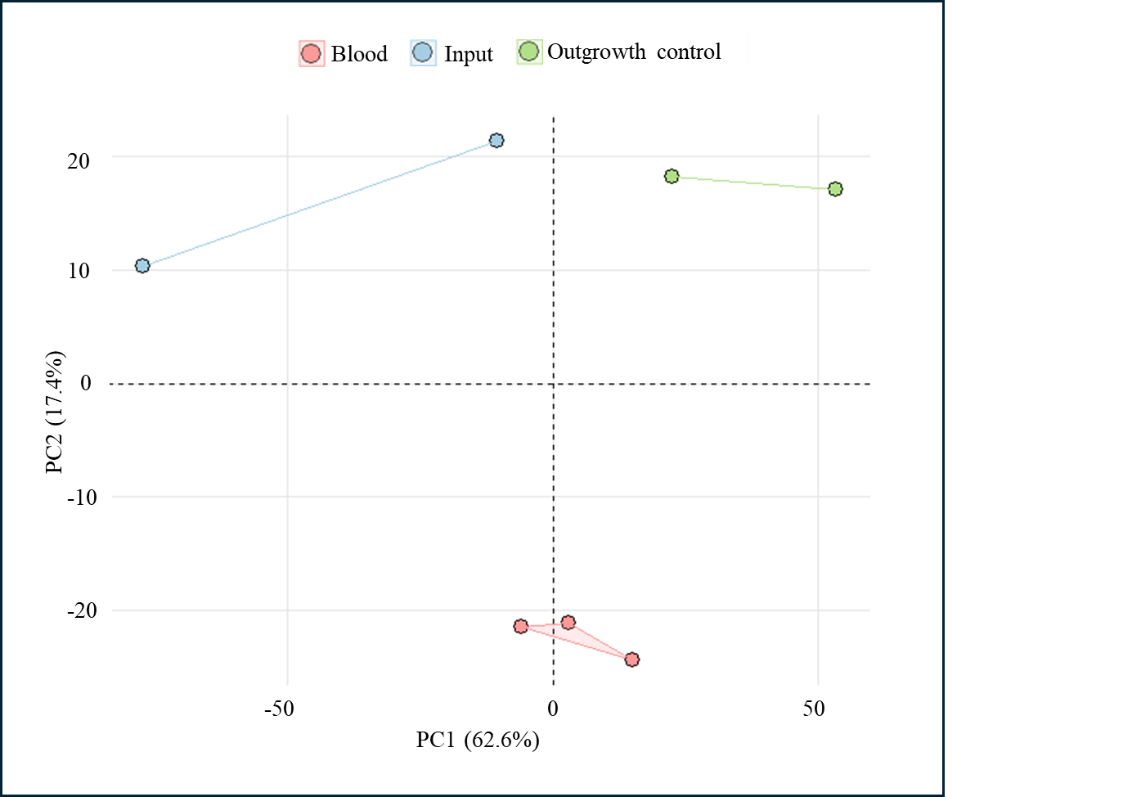


**Fig S1.** Principal Component Analysis of replicate variation across conditions. PCA was performed on normalized insertion counts for all genes to assess replicate similarity across input, outgrowth Control, and blood conditions. Replicates are represented as points colored by condition.

**Table S1.** TraDIS metrics for input, outgrowth, and output libraries.

^1^Number of mapped sequence reads against *S. aureus* USA300 JE2 genome (CP000255.1) (% of the raw data). ^2^ Number of Unique Insertions Sites (UISs). ^3^ The average distance between transposon insertions. ^4,5^ The initial transposon library (input) and the outgrowth controls (BHI) were obtained and analyzed previously (1, 2).


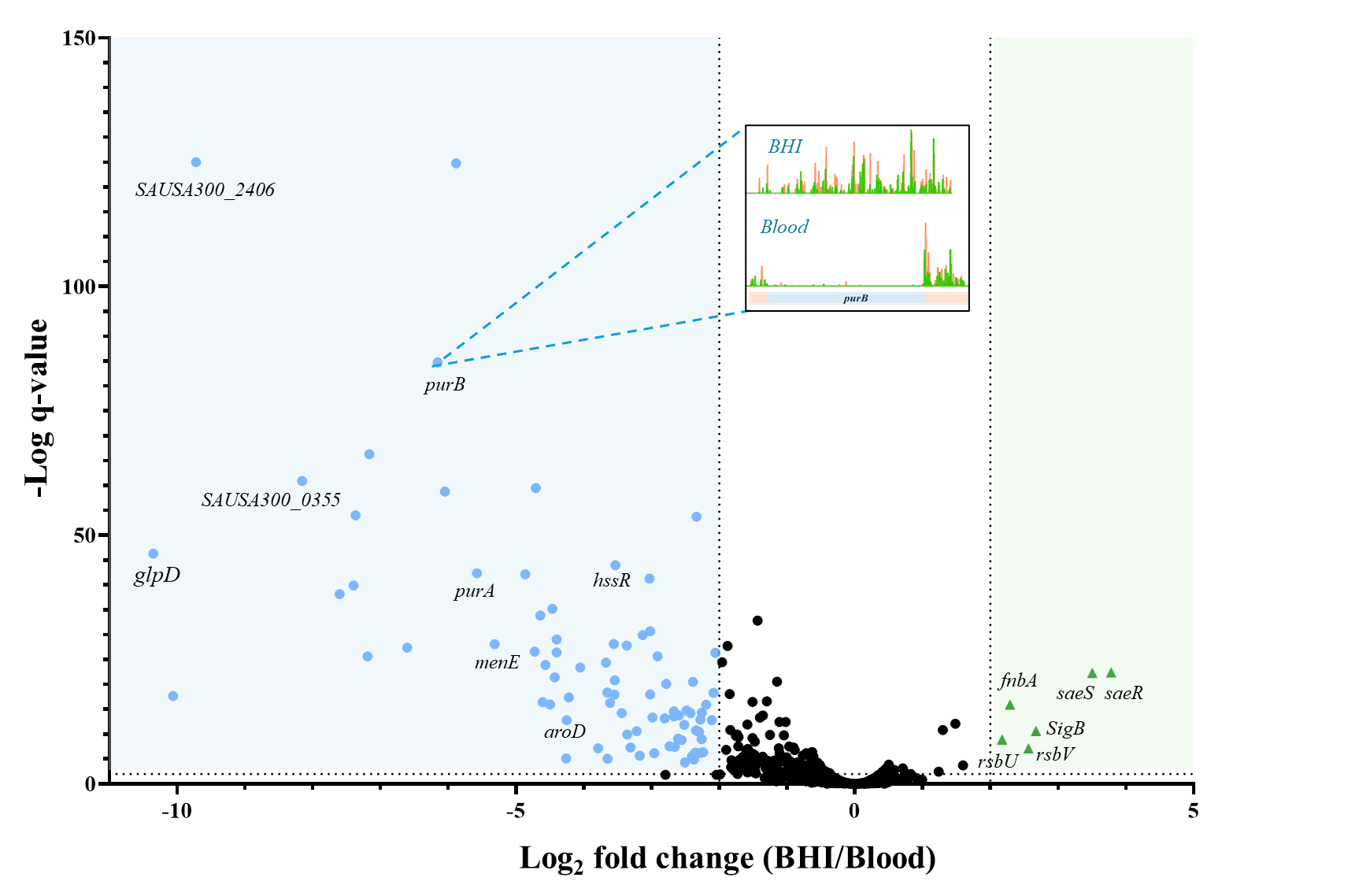


**Fig S2.** Genome-wide fitness determinants of *S. aureus* in human blood. Volcano plot of TraDIS data showing log₂ fold changes of transposon mutant abundance during growth in human blood compared with BHI. Genes are shown in blue for predicted fitness genes with (Log₂FC ≤ –2 and q-value < 0.01), and in green for enriched genes (Log₂FC > 2 and q-value < 0.01).

**Table S4.** Bacterial strains used in this study

| Strain | Description | Reference | |
| --- | --- | --- | --- |
| USA300 JE2 | USA300 CA-MRSA isolate cured of p01 and p03 plasmids / parent strain of the Nebraska Transposon Mutant Library | | (3) |
| NE529 (*purA*::Tn) | USA300 JE2 containing a transposon insertion in *purA*. Erm^r^ | | (3) |
| NE522 (*purB*::Tn) | USA300 JE2 containing a transposon insertion in *purB*. Erm^r^ | | (3) |
| NE233 (*glpD*::Tn) | USA300 JE2 containing a transposon insertion in *glpD*. Erm^r^ | | (3) |
| NE542 (*fbp*::Tn) | USA300 JE2 containing a transposon insertion in *fbp.* Erm^r^ | | (3) |
| NE1738 (*pncA*::Tn) | USA300 JE2 containing a transposon insertion in *pncA*. Erm^r^ | | (3) |
| NE1089 (*hssR*::Tn) | USA300 JE2 containing a transposon insertion in *hssR*. Erm^r^ | | (3) |
| NE1276 (*aroA2*::Tn) | USA300 JE2 containing a transposon insertion in *aroA2*. Erm^r^ | | (3) |
| NE1901 (*SAUSA300_0355*::Tn) | USA300 JE2 containing a transposon insertion in *SAUSA300_0355*. Erm^r^ | | (3) |
| NE1622 (*saeR*::Tn) | USA300 JE2 containing a transposon insertion in *saeR*. Erm^r^ | | (3) |
| NE186 (*fnbA*::Tn) | USA300 JE2 containing a transposon insertion in *fnbA*. Erm^r^ | | (3) |
| NE543 (*clfA*::Tn) | USA300 JE2 containing a transposon insertion in *clfA* Erm^r^ | | (3) |

**Table S5.** List of primers used in the TraDIS workflow

| Primer Name | Sequence (5'-3') | Reference |
| --- | --- | --- |
| SplA5-Top | G*AGATCGGTCTCGGCATTCCTGCTGAACCGCTCTTCCGATC*T | (4) |
| SplA5-Bottom | /5PHOS/G*ATCGGAAGAGCGGTTCAGCAGGTTTTTTTTTTCAAAAAAA*A | (4) |
| SplAP5.1 | C*AAGCAGAAGACGGCATACGAGATAACGTGATGAGATCGGTCTCGGCATTC*C | (4) |
| SplAP5.2 | C*AAGCAGAAGACGGCATACGAGATAAACATCGGAGATCGGTCTCGGCATTC*C | (4) |
| SplAP5.3 | C*AAGCAGAAGACGGCATACGAGATATGCCTAAGAGATCGGTCTCGGCATTC*C | (4) |
| ForwardTnL | CTTAAGTTTGCTTCGATGACTGG | (5) |
| Transposon-specific | AATGATACGGCGACCACCGAGATCTACACCTGAATTACCCTGTTATCCCTATTTAGGTGAC | (5) |
| Sequencing primer | GACACTATAGAAGAGACCGGGGACTTATCAGC | (5) |

**References**

1. Yousief SW, Abdelmalek N, Paglietti B. 2024. Optimizing phage-based mutant recovery and minimizing heat effect in the construction of transposon libraries in Staphylococcus aureus. Sci Rep 14:22831. https://doi.org/10.1038/s41598-024-73731-y.

2. Abdelmalek N, Yousief SW, Bojer MS, Alobaidallah MSA, Olsen JE, Paglietti B. 2025. The Secondary Resistome of Methicillin-Resistant Staphylococcus aureus to β-Lactam Antibiotics. Antibiotics 14:112. https://doi.org/10.3390/antibiotics14020112.

3. Fey PD, Endres JL, Yajjala VK, Widhelm TJ, Boissy RJ, Bose JL, Bayles KW. 2013. A Genetic Resource for Rapid and Comprehensive Phenotype Screening of Nonessential Staphylococcus aureus Genes. mBio 4:e00537-12. https://doi.org/10.1128/mbio.00537-12.

4. Barquist L, Mayho M, Cummins C, Cain AK, Boinett CJ, Page AJ, Langridge GC, Quail MA, Keane JA, Parkhill J. 2016. The TraDIS toolkit: sequencing and analysis for dense transposon mutant libraries. Bioinformatics 32:1109–1111. https://doi.org/10.1093/bioinformatics/btw022

5. Christiansen MT, Kaas RS, Chaudhuri RR, Holmes MA, Hasman H, Aarestrup FM. 2014. Genome-Wide High-Throughput Screening to Investigate Essential Genes Involved in Methicillin-Resistant Staphylococcus aureus Sequence Type 398 Survival. PLoS ONE 9:e89018. https://doi.org/10.1371/journal.pone.0089018.
